# Neuro-skeletal units and Smart Analgesia: reprogramming sensory neurons for fracture repair

**DOI:** 10.1016/j.mmr.2026.100044

**Published:** 2026-06-05

**Authors:** Long Bai, Jia-Can Su

**Affiliations:** a Organoid Research Center, Institute of Translational Medicine, Shanghai University, Shanghai 200444, China; b MedEng-X Institutes, Shanghai University, Shanghai 200444, China; c National Center for Translational Medicine (Shanghai) SHU Branch, Shanghai University, Shanghai 200444, China; d Wenzhou Institute of Shanghai University, Wenzhou, Zhejiang 325000, China; e Department of Orthopaedics, Xinhua Hospital Affiliated to Shanghai Jiao Tong University School of Medicine, Shanghai 200092, China

**Keywords:** Sensory neurons, Fracture repair, Smart Analgesia, Pain plasticity, Fibroblast growth factor 9 (FGF9)

Fracture repair is not simply a skeletal event, and has hematoma formation, inflammatory cell recruitment, vascular remodeling, periosteal progenitor activation, and callus maturation. The periosteum and marrow are richly innervated, particularly by sensory and sympathetic fibers, and these nerves are positioned to respond to pain with immune, vascular, and progenitor responses. Pain appears at the very beginning, yet it should not be treated only as an unpleasant readout of tissue damage. Persistent pain reflects maladaptive neuronal plasticity, in which channels such as Pannexin 1 (Panx1) can amplify adenosine triphosphate (ATP)-dependent excitability and hold sensory circuits in a chronic alarm state [1], [2]. At the same time, sensory nerve dysfunction, peripheral neuropathy, or denervation can impair bone regeneration and delay fracture repair [3]. Against this background, Xu *et al.* [4] provide a mechanistic link between sensory innervation and fracture repair. By mapping somatosensory afferents that innervate bone, they show that the same sensory system that detects fracture pain also provides trophic instructions required for repair. This dual role forms the basis of the neuro-skeletal unit (NSU) concept and reframes analgesia during fracture repair as a matter of selectively controlling pathological pain without interrupting sensory signals that support healing.

Recent work on the nerve-bone axis has placed sensory neurons within this network, with nerve growth factor/tropomyosin receptor kinase A (NGF/TrkA) signaling, calcitonin gene-related peptide (CGRP) release, neuroimmune coupling, and neurovascular regulation all emerging as relevant components. Xu *et al.* [4] extended this framework by asking three linked questions: which dorsal root ganglion (DRG) neurons project to bone, how these neurons respond after fracture, and which neuronal signals communicate back to the callus. Rather than treating skeletal innervation as a diffuse input, the authors identify a selective afferent population enriched for CGRP^+^ peptidergic nociceptors and Aβ-field low-threshold mechanoreceptors. After a fracture, bone-projecting DRG neurons do not merely increase injury-response genes. They enter a phased transcriptional program: early nociceptive and inflammatory activation, an intermediate secretory state marked by morphogens such as Transforming growth factor-β1 (TGF-β1), fibroblast growth factor 9 (FGF9), and sonic hedgehog (SHH), and a later return toward homeostasis. These changes are not only molecular signatures of injured neurons; they appear to shape the behavior of cells within the callus.

The main advantage of Xu *et al.* [4] moves beyond an anatomical description of skeletal innervation and identifies a defined sensory-neuron-derived repair signal. Among the identified factors, neuron-derived FGF9 stands out as a concrete regenerative signal that links the post-fracture sensory neuronal state to mesenchymal cell proliferation and osteogenic differentiation through fibroblast growth factor receptor (FGFR) signaling, which promotes mesenchymal cell proliferation and osteogenic differentiation, and therefore, as a plausible entry point for therapeutic development [5], [6]. In particular, this finding strengthens the NSU concept by providing a concrete molecular mechanism through which bone-projecting sensory neurons support callus formation.

The available evidence remains largely preclinical, and it is still unclear whether the same sensory neuronal programs operate in clinical conditions associated with impaired bone repair or altered peripheral nerve function [7]. In addition, the relative contributions of CGRP^+^ nociceptors and Aβ-field low-threshold mechanoreceptors to pain perception, vascular remodeling, immune regulation, and progenitor cell fate require further clarification. FGF9 is an important regenerative cue, but it is unlikely to represent the entire neuronal repair program; SHH, TGF-β-related pathways, CGRP, and immune mediators may act in concert during callus formation. These limitations highlight the need to distinguish maladaptive pain signaling from repair-supportive sensory activity during fracture healing. Future studies should determine whether analgesic interventions can relieve pathological pain while preserving sensory neuron-derived trophic signals that support callus formation.

Based on these findings, future models of fracture repair should no longer treat neural input as a peripheral variable. Instead, they should test how bone-projecting sensory neurons shape the callus at defined stages of healing. Spatial transcriptomics and single-cell approaches may help predict when FGF9-, SHH-, TGF-β-, or CGRP-related signals are most active, which callus cell populations respond to them, and whether these interactions can be selectively preserved during analgesic treatment [8]. Engineered systems pre-vascularized bone organoids and biomaterial constructs that include sensory neurons, endothelial networks, immune cells, and mesenchymal progenitors would allow researchers to test when neuronal signals are required, which target cells receive them, and which analgesic regimens spare repair [9], [10]. Such platforms could also model high-risk settings, including aging, neuropathy, chronic inflammation, and metabolic disease. In the longer term, fracture therapy may move beyond osteogenic stimulation alone toward neuro-vascular-immune orchestration: local delivery of FGF9 or morphogen combinations, restoration of sensory-neuron metabolic and epigenetic states, and analgesics that suppress maladaptive firing without erasing regenerative crosstalk.

In summary, Xu *et al.* [4] reposition fracture pain as part of a reparative circuit rather than a symptom divorced from healing. A remaining limitation is the lack of clear criteria for separating maladaptive pain signaling from repair-supportive neuronal activity. Defining this boundary will be essential for developing analgesic strategies that relieve pain without compromising bone repair.

## Abbreviations

ATP: Adenosine triphosphate

BMP: Bone morphogenetic protein

CGRP: Calcitonin gene-related peptide

DRG: Dorsal root ganglion

FGF9: Fibroblast growth factor 9

FGFR: Fibroblast growth factor receptor

NGF: Nerve growth factor

NSU: Neuro-skeletal unit

Panx1: Pannexin 1

SHH: Sonic hedgehog

TGF-β: Transforming growth factor-β1

TrkA: Tropomyosin receptor kinase A

## Ethics approval and consent to participate

Not applicable.

## Funding

This work was supported by the National Natural Science Foundation of China (82230071, 32471396, 82427809), the Shanghai Committee of Science and Technology (23141900600), the Shanghai Clinical Research Plan (SHDC2023CRT013), the Shanghai Municipal Demonstration Project for Innovative Medical Device Applications (23SHS05700), and the Young Elite Scientist Sponsorship Program by China Association for Science and Technology (YESS20230049).

## Data Availability

Not applicable.
